# Genome-Wide Gene Expression Effects of Sex Chromosome Imprinting in *Drosophila*

**DOI:** 10.1534/g3.113.008029

**Published:** 2013-12-06

**Authors:** Bernardo Lemos, Alan T. Branco, Pan-Pan Jiang, Daniel L. Hartl, Colin D. Meiklejohn

**Affiliations:** * Molecular and Integrative Physiological Sciences Program, Department of Environmental Health, Harvard School of Public Health, Boston, Massachusetts; † Department of Organismic and Evolutionary Biology, Harvard University, Cambridge, Massachusetts; ‡ Department of Ecology and Evolutionary Biology, Brown University, Providence, Rhode Island

**Keywords:** sex chromosome, imprinting, *Drosophila*, microarray

## Abstract

Imprinting is well-documented in both plant and animal species. In *Drosophila,* the Y chromosome is differently modified when transmitted through the male and female germlines. Here, we report genome-wide gene expression effects resulting from reversed parent-of-origin of the X and Y chromosomes. We found that hundreds of genes are differentially expressed between adult male *Drosophila melanogaster* that differ in the maternal and paternal origin of the sex chromosomes. Many of the differentially regulated genes are expressed specifically in testis and midgut cells, suggesting that sex chromosome imprinting might globally impact gene expression in these tissues. In contrast, we observed much fewer Y-linked parent-of-origin effects on genome-wide gene expression in females carrying a Y chromosome, indicating that gene expression in females is less sensitive to sex chromosome parent-of-origin. Genes whose expression differs between females inheriting a maternal or paternal Y chromosome also show sex chromosome parent-of-origin effects in males, but the direction of the effects on gene expression (overexpression or underexpression) differ between the sexes. We suggest that passage of sex chromosome chromatin through male meiosis may be required for wild-type function in F_1_ progeny, whereas disruption of Y-chromosome function through passage in the female germline likely arises because the chromosome is not adapted to the female germline environment.

Genomic imprinting refers to epigenetic marks placed on genes due to chromosomal transmission through the female and male germlines and often results in gene expression differences between maternally and paternally inherited alleles. Imprinting has been documented in insects, plants, mammals, and nematode worms (Anaka *et al.* 2009; Golic *et al.* 1998; Lloyd *et al.* 1999; Maggert and Golic 2002; Renfree *et al.* 2013; Sha and Fire 2005; Singh 1994) and is mediated by DNA methylation and histone modifications established during oogenesis and spermatogenesis (Sha 2008). In mammals, gynogenetic offspring are inviable (McGrath and Solter 1984; Thomson and Solter 1988), indicating that a balanced contribution of chromosomes of paternal and maternal origin is required for development in these lineages. Although the phenomenon is well-established in mammals, the extent of parent-of-origin effects on genome-wide gene expression is a matter of recent controversy (Babak *et al.* 2008; DeVeale *et al.* 2012; Gregg *et al.* 2010a, 2010b) because only ∼30 imprinted loci have been well-characterized and these are mostly found at a few clusters in the genome.

In contrast to mammals, the evidence for genome-wide parent-dependent allele-specific expression in *Drosophila melanogaster* is limited. First, both gynogenetic and androgenetic offspring are viable and fertile in *Drosophila* (Fuyama 1984; Komma and Endow 1995). Second, two recent surveys of allele-specific expression failed to identify evidence of parent-of-origin effects on gene expression in *Drosophila*, both among 24 genes assayed in *D. melanogaster* females (Wittkopp *et al.* 2006) and genome-wide in female *D. melanogaster/sechellia* F1 hybrids (Coolon *et al.* 2012). Nevertheless, complex patterns of gene expression inheritance have been documented in fruit flies, some of which are consistent with parent-of-origin effects (Gibson *et al.* 2004).

Most examples of imprinting in *Drosophila* involve epigenetic effects on heterochromatin that result from transmission through males or females and produce parent-of-origin effects on the expression of visible markers in or near the heterochromatic regions (Lloyd 2000; Maggert and Golic 2002). The link between imprinting and heterochromatin in *Drosophila* is supported by the observation that mutations in *Su(var)3-9*, a histone H3 methyl transferase, and heterochromatin protein 1 (HP1), a major component of heterochromatin, modify both imprinting and heterochromatin formation (Joanis and Lloyd 2002). Heterochromatic regions of the genome may be particularly sensitive to the differences in chromatin state and nuclear compaction that result from oogenesis and spermatogenesis (Fitch *et al.* 1998). Additionally, differences in heterochromatic content between male and female *Drosophila*, attributable to the presence of the 40-Mb heterochromatic Y chromosome in males, may lead to sex-specific gene regulation and chromosome conformation in both somatic and germline cells. The presence of Y-chromosome heterochromatin in males has been hypothesized to underlie differential sensitivity of male and female *Drosophila* to mutations affecting HP1 (Liu *et al.* 2005) and male-specific modification of heterochromatin by mutations affecting dosage compensation (Deng *et al.* 2009). Genetic differences in Y-linked heterochromatin content or satellite content have also been shown to have regulatory consequences for many hundreds if not thousands of autosomal and X-linked genes in males (Jiang *et al.* 2010; Lemos *et al.* 2008, 2010).

Various selective pressures have been proposed to explain the evolution of allele-specific expression or silencing (Brandvain *et al.* 2011; Haig 1997; Haig and Wilczek 2006; Rice *et al.* 2008; Wilkins 2010). In particular, paternal X chromosomes are transmitted solely to daughters. Hence, one route to achieve sex-specific expression would be through epigenetic marks placed on the X chromosome during spermatogenesis. Additionally, because X chromosomes are normally not transmitted from a father to his sons, epigenetic states imposed by spermatogenesis on X-linked chromatin could be incompatible with normal male germline function in the next generation. Similarly, because Y chromosomes are normally only found in males, they might acquire aberrant epigenetic states when passaged through the female germline.

To test for consequences of sex chromosome imprinting in *Drosophila*, we assayed genome-wide gene expression in adult *D. melanogaster* that have identical nuclear genotypes but differ only in the parent-of-origin of their X and Y chromosomes. We used compound X-X and X-Y chromosomes to generate three contrasts (Figure 1): males that inherit a maternal X chromosome and a paternal Y chromosome (X_M_Y_P_) *vs.* males that inherit a paternal X and a maternal Y (X_P_Y_M_); males that inherit a maternal compound X-Y chromosome and a paternal free Y (XY_M_Y_P_) chromosome *vs.* a paternal X-Y and a maternal free Y (XY_P_Y_M_); and females that inherit a paternal compound X-Y chromosome and a maternal free X chromosome (XY_P_X_M_) *vs.* females that inherit a paternal X-Y and a maternal X chromosome (XY_M_X_P_). All three contrasts compared flies that carry genetically identical sex chromosomes that differ only in their parent-of-origin, and they were tested in a common autosomal genetic background and under extensively controlled environmental conditions. We found very few genes differentially expressed between XY_P_X_M_ and XY_M_X_P_ females, consistent with other studies that found little evidence for genome-wide imprinting in *Drosophila* females (Coolon *et al.* 2012; Wittkopp *et al.* 2006), and we observed a significant negative correlation between imprinting effects on these few genes in males and females. However, we found that hundreds of genes were significantly differentially expressed in both male contrasts. Genes downregulated in adult male flies that inherit a paternal X chromosome and a maternal free Y chromosome (both X_P_Y_M_ and XY_P_Y_M_) are largely expressed specifically in the testes and genes upregulated in these flies are enriched for expression in the adult midgut. Because Y chromosomes are not normally found in females or inherited maternally, the epigenetic effects our experiments detect are not likely to be commonly observed in natural populations. Nonetheless, our results provide direct evidence that transmission of the sex chromosomes through the male or female germline results in differential epigenetic modification and demonstrates the consequences of these modifications for genome-wide gene expression.

**Figure 1:**
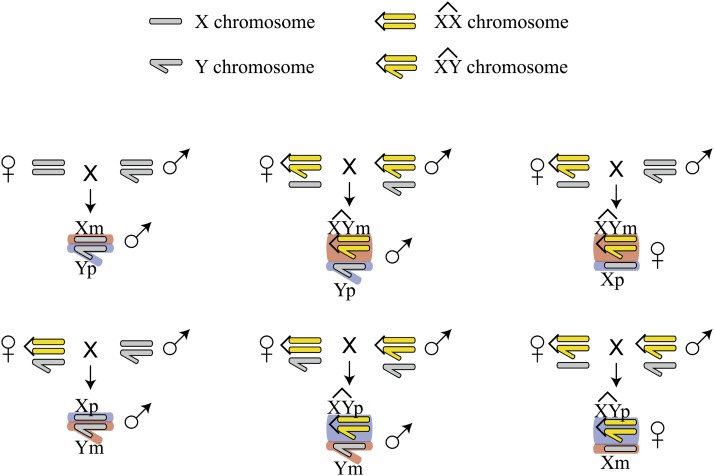
Crosses used to generate individuals with reversed sex chromosome parent-of-origin inheritance. Both free and attached X and Y chromosomes were substituted into a common autosomal background (Figure S1). Only the sex chromosomes are depicted for each cross. Hundreds of individual flies of each genotype were pooled for gene expression analysis.

## Materials and Methods

### Drosophila genetics and husbandry

To control the genetic background so that robust inferences regarding parent-of-origin effects on gene expression could be made, we precisely substituted an attached-X chromosome [C(1)M4,*y*^2^] and an attached X-Y chromosome [C(1;Y)3,In(1)FM7,*w*^1^,*m*^2^] into an inbred wild-type *D. melanogaster* genetic background (*Autw132*) using balancer and marked autosomes (Supporting Information, Figure S1). Following the generation of the C(1)M4,*y*^2^/ C(1;Y)3,In(1)FM7,*w*^1^,*m*^2^; *Aut*; *Aut* strain, crosses between this genotype and the parental *Autw132* strain were used to generate the experimental genotypes (Figure 1). We chose this approach rather than screening progeny for exceptional karyotypes resulting from nondisjunction, because it allowed careful control of the larval environment while simultaneously producing a large number of adult flies that allowed well-replicated microarray analysis. All three contrasts compared individuals carrying *Autw132* autosomes and identical sex chromosomes. Genetic constraints associated with our experimental approach required that the two male contrasts also differed in the maternally inherited cytoplasm (see *Discussion* for more details on this point). Both strains that provided maternal cytoplasm [C(1)M4,*y*^2^ and *Autw132*] were tested for the presence of *Wolbachia* by PCR using previously published primers and conditions (Montooth *et al.* 2010).

Both male and female fecundity differ substantially between attached-X females or attached X-Y males and *Autw132*; therefore, flies were reared to minimize variation in culture conditions and density between genotypes. All cultures were kept in a 25° incubator with a 12:12 light/dark cycle. To generate each genotype, 25 virgin males and 25 virgin females were placed together in vials with added yeast to initiate mating. Twenty-four hours later, two vials (*i.e.*, 50 males and 50 females) were combined into a mating cage on apple juice plates and live yeast paste to stimulate oviposition. Parents were transferred to a new laying cage 24 hr later, and again 48 hr later. On the third day after egg laying, larvae were removed from the apple juice plates by adding 20% sucrose solution and allowing the larvae to float to the surface. They were transferred by Pasteur pipette to PBS solution in a watch glass, and then 50 larvae were pipetted from the PBS onto a cotton plug. The plugs were then inserted into a vial of food without added yeast and the larvae were allowed to develop. Adults were collected as virgins, aged 2–3 days in single-sex vials, and then flash-frozen in liquid nitrogen at the same time of day to control for circadian effects on gene expression.

### Microarrays

Microarrays were constructed from 21,487 exon-specific PCR products amplified from the Oregon-R strain of *D. melanogaster* (Hild *et al.* 2003) that were spotted onto poly-L-lysine–coated slides (Thermo Scientific, Portsmouth, NH) using standard protocols (www.microarray.org). Total RNA was extracted from whole flies using TRIzol (Life Technologies). cDNA synthesis and labeling with fluorescent dyes (Cy3 and Cy5) as well as hybridization conditions were performed using 3DNA protocols and reagents (Genisphere, Hatfield, PA). For each genotype, total RNA was isolated from multiple replicates of ∼60 flies; each replicate was hybridized and scanned with an Axon 4000B scanner (Molecular Devices, Sunnyvale, CA) and GenePix Pro 6.0 software (Molecular Devices). Only microarray spots meeting the following quality control criteria were retained for further analysis: 70% of the foreground pixels within a spot had a signal intensity higher than 2 SDs above the median background signal intensity in at least one of the two channels (Cy3 or Cy5); the median foreground signal intensity was at least three-times as great as the median background signal intensity in at least one of the two channels; and a spot had more than 30 foreground pixels. Log2 ratios were normalized over spot signal intensity by loess smoothing using the *limma* package (Smyth 2004; Smyth and Speed 2003) and a span of 0.3. Spatial variation in signal intensity across arrays was removed by subsequently normalizing log2 ratios with loess smoothing over the physical location of the spots on the slide, using a span of 0.002, or approximately 250 spots.

### Experimental design and gene expression analysis

Gene expression differences between pairs of genotypes differing only in the parent-of-origin of the sex chromosomes were assayed by direct comparison of RNA samples on the spotted microarrays, incorporating dye swaps. For X/Y males and XY/X females, the experiments included two batches of independent RNA extractions and microarray hybridizations. We observed excellent agreement between the two batches for both male and female data and the two batches were combined for the final analysis. X_M_Y_P_ *vs.* X_P_Y_M_ males and XY_P_X_M_ *vs.* XY_M_X_P_ females were each contrasted on eight separate arrays. Limited emergence of XY/Y genotypes precluded the same level of replication for this genotype: the dataset in this case consists of five separate microarrays. The significance of differences in gene expression was assessed using a linear model in *limma* (Smyth 2004). Results were checked for consistency with the Bayesian analysis of gene expression levels (BAGEL) (Townsend and Hartl 2002), for which false discovery rates (FDRs) were empirically estimated by permutation of the dataset. Fold-change estimates showed remarkable concordance between *limma* and BAGEL (*ρ* = 0.99; *P* < 0.0001). The microarray gene expression data reported here can be obtained at the NCBI Gene Expression Omnibus database under accession number GSE51942.

### Bioinformatic analyses

Tissue specificity of gene expression was assessed using the FlyAtlas data (downloaded on August 2012) (Chintapalli *et al.* 2007). We filtered the data to include only the following nonredundant set of tissues: adult brain; adult accessory gland; adult crop; adult eye; adult fat body; adult hindgut; adult heart; adult midgut; adult salivary gland; adult thoracicoabdominal ganglion; ejaculatory duct; female spermathecaea; larval central nervous system; larval hindgut; larval midgut; larval salivary gland; larval trachea; larval malpighian tubules; ovary; and testes. For each Affymetrix probe in the FlyAtlas dataset and for each tissue, we arbitrarily set expression level of probes with “absent” calls to 1. When multiple probe sets matched a single gene, the probe set with the strongest signal intensity across all tissue samples was chosen and the redundant probe sets were discarded. Tissue-specific expression was determined using the *τ* metric (Yanai *et al.* 2005) and a cutoff of *τ* > 0.9. Chromatin status was determined from previously published genome-wide protein binding and covalent histone modification profiles summarized into a five-state model (chromatin “colors”) (Filion *et al.* 2010). Only genes in which a single chromatin state covered the entire coding region were assigned a chromatin color. All analyses were performed in R (version 2.15.2) (R Core Team 2011). Microarray analyses used the *limma* package (version 3.14.4) (Smyth 2004); bivariate regression analysis used standard major axis regression with the *smatr* package (version 3.2.4).

## Results

### Abundant genome-wide expression effects in X_P_Y_M_ adult males

Our experiments directly compared genome-wide patterns of gene expression between identical genotypes that differ in the parent-of-origin of the X and Y chromosome. The crossing schemes used to generate the parental genotypes are shown in Figure S1, and the crosses used to generate the experimental individuals are shown in Figure 1. For all three contrasts shown in Figure 1, gene expression differences were assayed in individuals with precisely the same nuclear genotype, so our results are not confounded by variation in the genetic background of the contrasted individuals. We first assayed gene expression in samples of adult male *D. melanogaster* with a standard X/Y sex chromosome karyotype that differed solely in the parental origin of the X and Y chromosomes [typical X-maternal / Y-paternal (X_M_Y_P_) flies *vs.* X-paternal / Y-maternal (X_P_Y_M_) flies]. At a FDR of 0.05, we identified 905 genes significantly differentially expressed between X_M_Y_P_ and X_P_Y_M_ individuals. This large number of differentially expressed genes was confirmed by a second independent batch of arrays, which resulted in 898 genes differentially expressed as a consequence of X-chromosome and Y-chromosome inheritance through the female or male germline. More than 60% of the genes identified as differentially expressed in the first experiment were replicated in the second set of arrays, and we found a highly significant concordance in fold-change estimates for gene expression variation between the two experiments (*ρ* = 0.81; *P* < 0.0001). Together, these results indicate a substantial effect on gene expression in adult male *D. melanogaster* resulting from X-chromosome and Y-chromosome parent-of-origin. Because of the strong concordance between the two array batches, we combined them into a single analysis, which substantially increased statistical power. This resulted in the identification of 2535 genes differentially expressed between X_M_Y_P_ and X_P_Y_M_ males at FDR < 0.05. In all subsequent analyses, we focused on this combined dataset.

Imprinting effects are generally mediated by epigenetic modifications that modulate expression via *cis*-regulation. The gene expression changes we observe could be the direct consequence of such *cis*-regulatory control, or a downstream consequences of such modifications. In the former case, we would expect differentially expressed genes to be located primarily on the X or the Y chromosome, whereas in the latter case differentially expressed genes might be dispersed throughout the genome. The number of differentially expressed genes located on the major autosomal arms or the small heterochromatic fourth chromosome is similar to random expectation (Table 1), and we observed a minor but significant deficit of differentially expressed genes on the X chromosome (17% of genes detected on the array are X-linked, 15% of differentially expressed genes are X-linked; χ^2^ test; *P* = 0.007). The slight but significant enrichment of differentially expressed genes on chromosome arm 2L (Table 1) likely resulted from the effects we observed on genes that are expressed specifically in the testis, which are overrepresented on 2L (χ^2^ = 13.6; *P* = 0.0002). Thus, there is little evidence for direct expression effects on X-linked genes as a consequence of X chromosome transmission through the male or the female germline, supporting that conclusion that the observed gene expression differences are downstream consequences of epigenetic modifications to the sex chromosomes.

**Table 1:** Chromosomal location of significantly differently expressed genes

	X	2L	2R	3L	3R	4	Other*^a^*	Total
All spots	2719	2992	3225	3235	3915	98	514	16,698
	16.8%	18.5%	19.9%	20.0%	24.2%	0.6%		
X/Y males	**366**	**505**	518	511	571	9	55	2535
	**14.8%**	**20.4%**	20.9%	20.6%	23.0%	0.4%		
XY/Y males	**21**	**63**	52	55	63	1	11	266
	**8.2%**	**24.7%**	20.4%	21.6%	24.7%	0.4%		
XY/X females	**0**	11	11	8	8	0	3	41
	**0.0%**	28.9%	28.9%	21.1%	21.1%	0.0%		

Numbers in bold indicate a significantly different proportion from that expected (χ^2^ test, FDR = 0.05).

a Microarray probes that are not mapped to the assembled *D. melanogaster* reference genome.

### Testis-specific genes are downregulated in X_P_Y_M_ males

**Figure 2:**
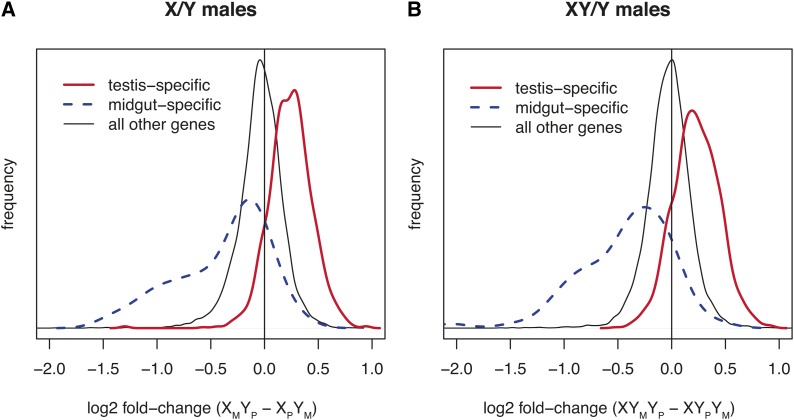
Tissue-specific expression effects of reversed parent-of-origin sex chromosome inheritance. Shown are the distributions of parent-of-origin effects for genes expressed specifically in the testes, the adult midgut, and all other genes. (A) Log2 expression differences between X_M_Y_P_ and X_P_Y_M_ males. The median fold-change of all three groups of genes is significantly different from zero (testis-specific genes = 0.24; midgut-specific genes = −0.23; all other genes = −0.03; *P*_MW_ < 0.0001 in all three cases). (B) Log2 expression differences between XY_M_Y_P_ and XY_P_Y_M_ males. The median fold-change of all three groups of genes is significantly different from zero (testis-specific genes = 0.22; midgut-specific genes = −0.36; all other genes = −0.02; *P*_MW_ < 0.0001 in all three cases).

To determine whether genes differentially expressed as a result of sex chromosome parent-of-origin share a common organismal expression pattern, we compared our results with microarray data from dissected larval and adult organs and tissues (Chintapalli *et al.* 2007) (Figure S2). We observed that genes expressed specifically in the testis (see *Materials and Methods*) were significantly overrepresented among genes significantly differently expressed between X_M_Y_P_ and X_P_Y_M_ males (12% of genes detected on the array were testis-specific; 32% of differentially expressed genes were testis-specific; Fisher exact test, *P*_FET_ < 0.0001). Additionally, there was a severe bias among testis-specific genes toward downregulation in X_P_Y_M_ males relative to X_M_Y_P_ males; 575/585 (98%) of significantly differently expressed testis-specific genes were downregulated in males inheriting a paternal X chromosome and a maternal Y chromosome (*P*_FET_ < 0.0001). Furthermore, the magnitude of differential expression among testis-specific genes, regardless of statistical significance, suggested that downregulation in X_P_Y_M_ males was common to testis-specific genes as a group (Figure 2). The median log2 expression difference between X_M_Y_P_ and X_P_Y_M_ males among testis-specific genes was 0.24 (1.18-fold; Mann-Whitney test, *P*_MW_ < 0.0001).

We observed a pattern complementary to that seen for testis-specific genes among genes expressed specifically in the adult midgut (Figure S2); of the 71 adult midgut-specific genes assayed on the arrays, 34 (48%) were significantly upregulated in X_P_Y_M_ males (*P*_FET_ < 0.0001), whereas only one adult midgut-specific gene was significantly downregulated in X_P_Y_M_ males. Similarly, upregulation in X_P_Y_M_ males was common to adult midgut-specific genes as a group (Figure 2); the median log2 expression difference between X_M_Y_P_ and X_P_Y_M_ males for all adult midgut-specific genes was −0.23 (0.85-fold; *P*_MW_ < 0.0001). In contrast to testis-specific and midgut-specific genes, all other genes assayed showed no strong bias among expression differences between X_M_Y_P_ and X_P_Y_M_ males (Figure 2 and Figure S2).

### Gene expression effects in XY_P_Y_M_ males recapitulate those in X_P_Y_M_ males

The comparison between X_M_Y_P_ and X_P_Y_M_ males did not allow us to distinguish whether the effects we observed resulted from reversed parent-of-origin of the X chromosome, the Y chromosome, or both. To further dissect the consequences of sex chromosome transmission on gene expression, we compared a second pair of male genotypes that carry a compound X-Y chromosome as well as a free Y chromosome. Using a similar crossing scheme as that used for the X_M_Y_P_ *vs.* X_P_Y_M_ contrast (Figure 1), we compared males that differ solely in the parent-of-origin of the compound X-Y and the free Y chromosome (XY_M_Y_P_ *vs.* XY_P_Y_M_). Males of these genotypes inherit both a paternal and a maternal Y chromosome; differences in gene expression between these genotypes must therefore result from either parent-of-origin effects associated with the X chromosome or different parent-of-origin effects associated with the Y-linked component of the compound X-Y *vs.* the free Y chromosome. We detected substantially fewer gene expression differences in XY/Y males than we observed in X/Y males; only 266 genes were significantly differently expressed between XY_M_Y_P_ and XY_P_Y_M_ males at FDR = 0.05. However, the XY/Y genotypes were assayed at a level of replication (see *Materials and Methods*) equivalent to a single batch of the X/Y experiments and showed substantially lower statistical power than the combined X/Y dataset (Figure S3).

**Figure 3:**
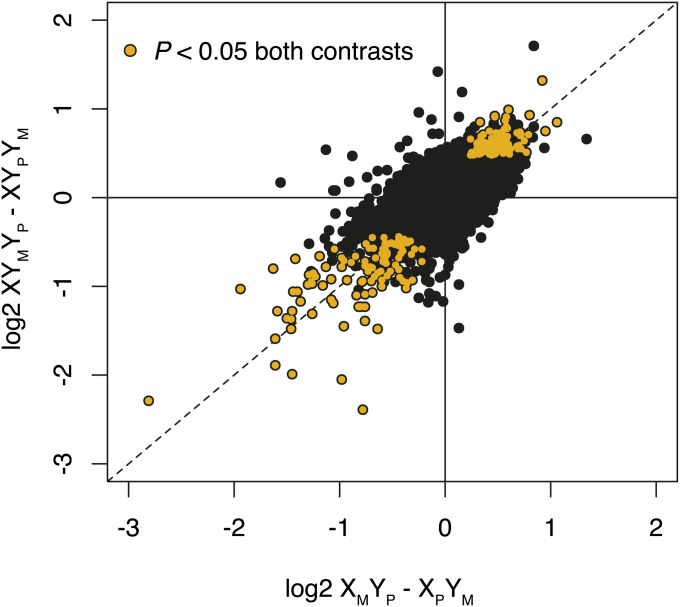
Gene expression differences in X/Y males and XY/Y males with reversed sex chromosome parent-of-origin inheritance. Orange points indicate genes significantly differently expressed between both X_M_Y_P_ *vs.* X_P_Y_M_ and XY_M_Y_P_ *vs.* XY_P_Y_M_ males. The two experiments are highly significantly correlated (*P* < 0.0001), both for all genes (*ρ* = 0.68) and the set of 205 differentially expressed genes common to both the XY/Y and X/Y experiments (*ρ* = 0.85). Dashed line has a slope of one.

We compared the list of genes significantly differently expressed in X/Y and XY/Y males and found that 77% (205 genes) of the genes differently expressed between XY_M_Y_P_ and XY_P_Y_M_ males at an FDR of 0.05 were also differently expressed between X_M_Y_P_ and X_P_Y_M_ males. Furthermore, fold-change estimates across all genes were remarkably similar in the XY/Y and the X/Y comparisons (*ρ* = 0.68; *P* < 0.0001) (Figure 3), with a stronger correlation in the set of 205 differentially expressed genes common to both the XY/Y and X/Y experiments (*ρ* = 0.85; *P* < 0.0001). None of these 205 genes had sign differences in fold-change estimates between the X/Y and XY/Y experiments. These observations indicate that the gene expression effects attributable to paternal inheritance of the X chromosome or maternal inheritance of a free Y chromosome are similar in X/Y and XY/Y males.

The biases observed in X/Y males toward upregulation and downregulation among tissue-specific genes are also detectable in XY/Y males. Testis-specific genes are significantly enriched among genes downregulated in XY_P_Y_M_ males relative to XY_M_Y_P_ males (57% of genes downregulated in XY_P_Y_M_ males are testis-specific; *P*_FET_ < 0.0001) and significantly depleted among genes significantly upregulated in XY_P_Y_M_ males (0/89 genes upregulated in XY_P_Y_M_ males are testis-specific; *P*_FET_ < 0.0001). Adult midgut-specific genes are enriched among genes upregulated in XY_P_Y_M_ males (0.8% of genes detected on the arrays are adult midgut-specific; 9.4% of genes upregulated in XY_P_Y_M_ males are adult midgut-specific, *P*_FET_ < 0.0001). As is the case with X/Y males, testis genes as a group are downregulated and adult midgut-specific genes as a group are upregulated in males inheriting a paternal X and maternal free Y compared to males inheriting a maternal X and paternal free Y chromosome (Figure 2).

### Somatic upregulation of the X chromosome in X_P_Y_M_ males

In *D. melanogaster* males, expression from the hemizygous X chromosome is globally upregulated by the male-specific lethal (MSL) complex to compensate for the difference in X *vs.* autosomal dosage between males and females (Lucchesi *et al.* 2005). The MSL complex contains chromatin-remodeling proteins and two X-linked noncoding RNAs, *roX1* and *roX2*, whose transcription facilitates localization of the MSL complex to the X chromosome (Meller *et al.* 1997). As the name suggests, mutations in genes that encode components of the MSL complex are generally lethal in males and have few effects in females. However, male *roX1 roX2* mutants that inherit a maternal Y chromosome have substantially higher survival than *roX1 roX2* males inheriting a paternal Y (Menon and Meller 2009), whereas there is no effect of Y-chromosome imprinting on viability in males with wild-type dosage compensation. One potential mechanism underlying this observation is upregulation of the X chromosome in the presence of a maternal Y chromosome (Menon and Meller 2009). Supporting this idea, we observed significantly more X-linked than autosomal genes upregulated in X_P_Y_M_ males (Table 2), and the magnitude of this excess was stronger among genes located in chromatin domains characterized by a histone modification (H3K36me3) that has been shown to recruit the MSL complex (Filion *et al.* 2010; Larschan *et al.* 2007). This excess of upregulated X-linked genes is not observed among testis-specific genes (Table 2) (*P*_FET_ = 0.372), which is consistent with the hypothesis that X-chromosome dosage compensation is absent from the male germline (Meiklejohn *et al.* 2011; Rastelli and Kuroda 1998). In *roX1 roX2* mutants, enhanced viability attributable to a maternal Y chromosome is masked by the simultaneous presence of a paternal Y chromosome (Menon and Meller 2009). We also observed gene expression effects consistent with this masking in the XY/Y male comparison, in which, in contrast to X/Y males, there is no difference in the proportion of upregulated genes on the X chromosome and the autosomes (data not shown).

**Table 2:** Somatic upregulation of X-linked genes in X_P_Y_M_ males

					Excluding Testis-Specific and Midgut-Specific Genes*^a^*			
	All Genes		Testis-Specific Genes				YELLOW Genes*^b^*	
	X-linked	Autosomal	X-linked	Autosomal	X-linked	Autosomal	X-linked	Autosomal
X_M_Y_P_ > X_P_Y_M_	161	1103	80	495	38	308	10	126
X_M_Y_P_ < X_P_Y_M_	205	1002	0	10	141	722	57	265
Odds ratio	0.71		—		0.63		0.37	
*P*	0.0032		NS		0.0196		0.0036	

NS, not significant.

a Includes only genes with FlyAtlas expression data.

b Euchromatic genes enriched for H3K36me3 (Filion *et al.* 2010).

### Little effect of Y chromosome parent-of-origin on gene expression in XY/X female genotypes

To investigate further the effects of Y-linked inheritance on global gene expression, we contrasted female genotypes that carry paternal or maternal Y chromosomes (see *Materials and Methods*). As with the male comparisons, we compared two XY/X female genotypes with the same nuclear genotype but that differed in the parent-of-origin of a single free X chromosome and a compound XY chromosome (XY_M_X_P_ *vs.* XY_P_X_M_) (Figure 1). Although this contrast between female genotypes was performed at a level of replication equivalent to that of the X/Y male comparison (*Materials and Methods*), at an FDR of 0.05 we detected only 41 genes differently expressed between XY_M_X_P_ and XY_P_X_M_ females (Figure S3 and Table 1). This suggests that transmission of the Y chromosome through the male or female germline has a small impact on global gene expression in females. None of the genes differentially expressed between XY_M_X_P_ and XY_P_X_M_ females are X-linked (Table 1), whereas 17% of genes robustly expressed on the female arrays are X-linked (*P*_FET_ = 0.002), suggesting that, as in males, parent-of-origin expression effects in females are indirect and downstream consequences of epigenetic modifications to sex chromosome chromatin.

**Figure 4:**
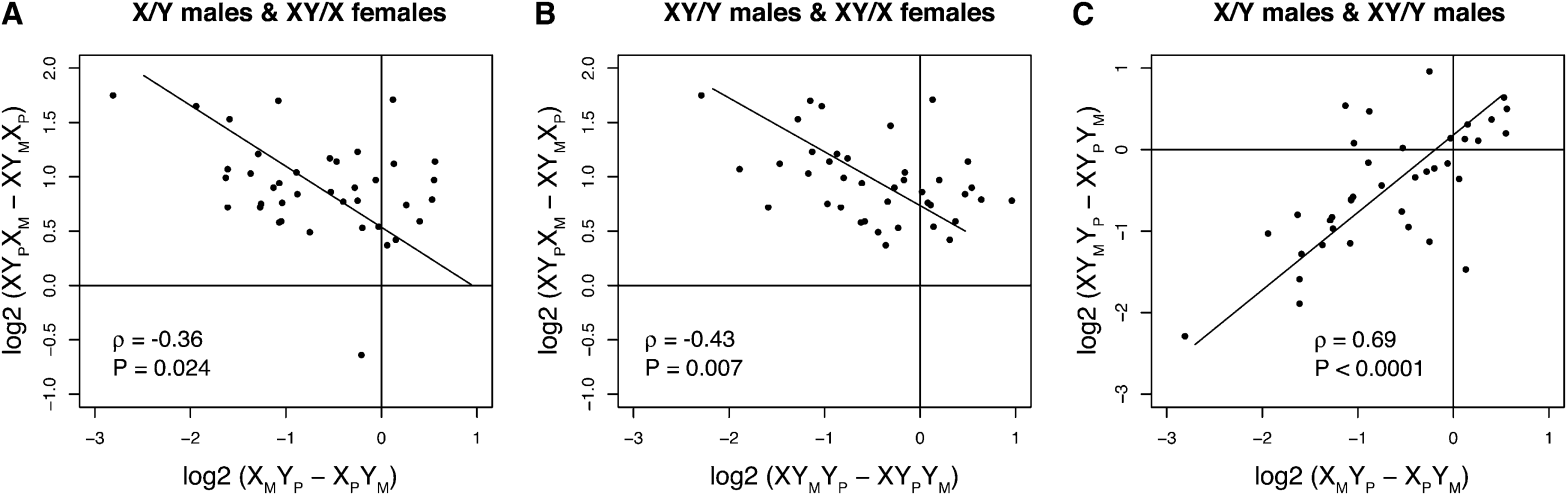
Expression differences in males and females for genes significantly differently expressed between females with reversed sex chromosome parent-of-origin inheritance. (A) Expression in X/Y males and XY/X females. There is a marginally significant negative correlation between gene expression effects attributable to parent-of-origin of the free X chromosome or the Y chromosome in males *vs.* females. (B) Expression in XY/Y males and XY/X females. There is a significant negative correlation between parent-of-origin effects attributable to the free Y chromosome in males and the attached X-Y chromosome in females. (C) Expression in X/Y and XY/Y males. There is a highly significant positive correlation attributable to parent-of-origin effects on the X chromosome or the free Y chromosome between these two male genotypes.

There is substantial overlap between genes differentially expressed in the female contrast and both male contrasts—62% and 46% of genes significant in females are also significant in X/Y males and XY/Y males, respectively (*P*_FET_ < 0.0001 in both cases). Furthermore, among those genes significantly differentially expressed between XY_M_X_P_ and XY_P_X_M_ females, we observed a negative correlation between the expression effects resulting from parent-of-origin of the compound XY chromosome in females and the free Y chromosome in males (Figure 4). This strong overlap and negative correlation between the sexes suggest that differential expression of these genes may result from the same regulatory mechanism in males and females and that this mechanism leads to the opposite effect (upregulation *vs.* downregulation) in males and females.

## Discussion

Imprinting in *Drosophila* has been detected via differential expression of visible markers when transmitted through sperm or eggs, such as those on the rearranged *Dp*(1*;f*)*LJ*9 mini-X chromosome (Anaka *et al.* 2009) and *P*-element insertions on the Y chromosome (Haller and Woodruff 2000; Maggert and Golic 2002). Imprinting of the *Drosophila* Y chromosome has also been shown to partially suppress mutations affecting dosage compensation of the X chromosome in males (Menon and Meller 2009). Here, we report extensive genome-wide expression differences resulting from sex chromosome transmission through the male *vs.* female germlines. Our results extend previous observations that Y-chromosome transmission through the egg or the sperm leads to differential epigenetic modification of the Y (Golic *et al.* 1998; Lloyd 2000; Maggert and Golic 2002) and indicates that such modifications influence genome-wide gene expression in whole adult males.

We observed an enrichment of testis-specific genes differentially regulated by sex-chromosome parent-of-origin effects, and these effects are strongly biased toward downregulation in males inheriting a paternal X and a maternal free Y chromosome, regardless of whether they also inherit the Y-linked component of an attached X-Y chromosome from their father. Genes expressed specifically in the midgut show complementary patterns to those specific to the testis—adult midgut genes are overrepresented among genes significantly upregulated in X_P_Y_M_ and XY_P_Y_M_ males and are upregulated as a group in these genotypes (Figure 2). The median magnitude of differential expression among testis-specific and midgut-specific genes is small (0.78-fold to 1.18-fold), consistent with the hypothesis that the effects of sex chromosome imprinting are dispersed throughout the genome in these cell types. However, by conducting expression assays on whole animals, we have likely underestimated the magnitude of the expression effects we observed on genes expressed only in a subset of cell types (Chintapalli *et al.* 2007).

In contrast to the large number of genes differentially expressed in males as a result of parent-of-origin of the sex chromosomes, we detected only 41 genes that were significantly differentially expressed between female genotypes inheriting a maternal Y chromosome (XY_M_X_P_) *vs.* those inheriting a paternal Y chromosome (XY_P_X_M_). Nonetheless, we saw a strong and statistically significant correlation between shared parent-of-origin effects in X/Y and XY/Y males and in XY/X females (Figure 4), suggesting that expression of these few genes was influenced by transmission of a Y chromosome through the male or female germline, regardless of the sex of the progeny. The direction of the expression effect (upregulation or downregulation) was reversed between males and females, with 39/41 genes showing greater expression levels in females inheriting a maternal Y chromosome and 30/41 showing reduced expression in males inheriting a maternal Y chromosome.

Although we do not know the molecular nature of the imprint placed on the sex chromosomes that led to these effects on gene expression, we suggest that passage through the male or female germline produces differences in the chromatin state of the heterochromatic regions of the sex chromosomes, such as the degree of heterochromatinization or nature of chromatin packaging. Previous associations between heterochromatin and imprinting in *Drosophila* (Lloyd 2000) suggest that these regions of the genome are normally differentially packaged in oogenesis and spermatogenesis; the indirect and *trans*-regulatory effects we detected likely resulted from these effects on the substantial amount of heterochromatin present in the 40-Mb Y-chromosome. This, in turn, could differentially sequester heterochromatin-binding proteins such as HP1 and Su(var)3-9, leading to differential expression of genes throughout the genome whose expression is affected by the amount of available HP1 (Zuckerkandl 1974).

### Are parent-of-origin effects attributable to the X or the Y chromosome?

By itself, the contrast between X_M_Y_P_ and X_P_Y_M_ males did not allow us to determine whether the effects on gene expression or testis size were attributable to reversed parent-of-origin of the X or the Y chromosome. The strong concordance between the X/Y and XY/Y contrasts, however, suggests that these effects may not be solely ascribed to Y-chromosome imprinting, because XY_M_Y_P_ and XY_P_Y_M_ males simultaneously inherit both a paternal and maternal Y chromosome. One possible explanation for our observations is that these effects result from transmission of the free Y chromosome through the male *vs.* female germlines and that in XY/Y males, these effects are not masked or compensated by the presence of the Y-linked complement of the compound XY chromosome inherited from the other parent. The compound XY chromosome we used here [*C*(1*;Y*)3, *In*(1)*FM7*, *w*^1^, *m*^2^] contains a full inversion of the X chromosome and may have other structural rearrangements or aneuploidy for X-linked or Y-linked heterochromatin, which could lead to differences in parent-of-origin effects between this chromosome and the free Y. An alternative possibility is that the changes in gene expression in both X/Y and XY/Y males result from parent-of-origin effects associated with the X chromosome. In addition to more than 2500 X-linked protein-coding genes located in 20 Mb of euchromatic DNA, the *Drosophila* X chromosome contains ∼20 Mb of heterochromatic DNA. Epigenetic modification of X-linked heterochromatin resulting from transmission through spermatogenesis *vs.* oogenesis could have effects on gene expression similar to those proposed for the Y chromosome. X-linked protein-coding genes are underrepresented among genes differentially expressed in our contrasts (Table 1), consistent with the hypothesis that such epigenetic modification of the X chromosome would be concentrated in heterochromatic regions and that this modification affects gene expression genome-wide in *trans*.

### Parent-of-origin effects in males are concentrated in the testes

Genes expressed specifically in the male germline are disproportionately affected by epigenetic consequences of X-chromosome or Y-chromosome passage through parental male or female germlines. The *Drosophila* Y chromosome contains at least 14 single-copy protein-coding genes, all of which are expressed only in the testes, and it is dispensable for both sex determination and male somatic development (Carvalho *et al.* 2009; Marsh and Wieschaus 1978). Despite its small coding potential, the Y chromosome comprises 40 Mb of DNA, and Y-linked variation has been shown to have widespread effects on gene expression (Jiang *et al.* 2010; Lemos *et al.* 2008, 2010), presumably via modulation of global chromatin status. Although genes regulated by differences between Y chromosomes are not restricted to the germline, in some experiments male-biased and testis-specific genes are disproportionately affected by Y-linked regulatory variation (Branco *et al.* 2013; Lemos *et al.* 2008; Sackton *et al.* 2011).

One interpretation of these patterns is that sex chromosome imprinting affects the expression of genes that share a common regulatory feature and that testis-specific and midgut-specific genes are overrepresented among this group of genes. A comparison of our results with genome-wide analyses of chromatin-binding protein occupancy and covalent histone modifications in *Drosophila* cell culture (Filion *et al.* 2010) indicates that genes regulated by sex chromosome imprinting are preferentially located in repressive chromatin domains in Kc167 cells; however, this can be explained by the enrichment of all tissue-specific genes in these repressive domains (Table S1). The global shifts observed among all testis-specific and midgut-specific genes (Figure 2) suggest an alternative interpretation—sex chromosome imprinting may influence genome-wide gene expression in these cell types. This interpretation is supported by patterns of expression among genes whose expression is *not* specific to the testes or adult midgut. We observed significant correlations between testis enrichment (a quantitative measure of expression level in the testis *vs.* other cell types; see *Materials and Methods*), midgut enrichment, and parent-of-origin effects on differential expression (Figure S4) among all genes not expressed solely in these two cell types. Specifically, there is a significant positive relationship between testis-enrichment and differential expression in both male contrasts (*ρ* = 0.31 and *P* < 0.0001 in X/Y males; and *ρ* = 0.23 and *P* < 0.0001 in XY/Y males); there is a significant negative relationship between midgut enrichment and differential expression in both male contrasts (*ρ* = −0.13 and *P* < 0.0001 in X/Y males; *ρ* = −0.19 and *P* < 0.0001 in XY/Y males). Neither testis enrichment nor midgut enrichment showed a significant relationship with differential expression in females (*P* = 0.99 and *P* = 0.84, respectively). This indicates that these global expression effects in the testis and midgut are limited to males. Further experiments may determine if sex chromosome imprinting leads to differences in the anatomy of the testis or midgut that could contribute to the expression effects we observed among genes specific to these tissues.

Sex chromosome imprinting could directly modify expression of testis-specific genes by changing the amount of available chromatin-associated proteins such as HP1, but it is also possible that imprinting effects on master transcriptional regulators in the male germline could have downstream consequences on the expression of testis-specific genes. For example, a segment of the Y chromosome thought only to contain repetitive sequences acts as a *trans*-activator of gene expression specifically in the testes (Zhang *et al.* 2000); epigenetic modification of this segment could affect the expression of many genes. However, as noted, the effects observed in XY/Y males indicate that a model involving imprinting of the Y chromosome requires that the parental imprint placed on the free Y chromosome is dominant in its effects over the parental imprint on the Y-linked segment of the compound X-Y chromosome. Although there are examples of imprinting involving X chromosome heterochromatin (Lloyd 2000), we are not aware of any reported associations between this region of the genome and regulation of gene expression in the male germline, with the possible exception of the *Stellate* heterochromatic repeats (Egorova *et al.* 2009; Palumbo *et al.* 1994a, 1994b).

### Cytoplasmic effects on gene expression

Although all the genotypes we contrasted carried identical X and Y chromosomes and autosomes, it was not possible to similarly control the source of the maternal cytoplasm in our crosses. This is because, in our crossing scheme, maternal inheritance of a Y chromosome requires a compound-X chromosome, and compound-X chromosomes co-segregate with maternal cytoplasm, precluding the introduction of a different cytoplasm into the *C*(1)*M4,y*^2^ compound-X strain, or the introduction of the *C*(1)*M4,y*^2^ cytoplasm into another X-chromosome genotype. Thus, both X_M_Y_P_ and XY_M_Y_P_ males inherited the *Autw132* strain cytoplasm, whereas the X_P_Y_M_ and XY_P_Y_M_ males inherited cytoplasm from the *C*(1)*M4,y*^2^ strain. The two female genotypes we contrasted share the same cytoplasm and mtDNA (*Autw132*), and so the small number of genes differentially expressed between XY_P_X_M_ and XY_M_X_P_ females cannot be attributable to cytoplasmic effects. The intracellular endosymbiotic bacterium *Wolbachia* can have profound effects on host fitness, physiology, and development (Clark *et al.* 2005; Ikeya *et al.* 2009; Mercot and Charlat 2004); however, neither cytoplasm used in these experiments carries *Wolbachia* (data not shown) (Montooth *et al.* 2010).

There are conflicting results in the literature regarding the contribution of cytoplasmic effects to genome-wide gene expression in *Drosophila*. Although classic quantitative genetic experiments detected negligible cytoplasmic contribution to expression variation in *D. simulans* (Wayne *et al.* 2007), a recent study identified cytoplasmic effects on genome-wide gene expression in *D. melanogaster* and found that these effects were stronger in males and included many testis-specific genes (Innocenti *et al.* 2011). One possibility, then, is that the patterns of differential gene expression we observed were caused by cytoplasmic factors, such as mtDNA variants, that led to differential gene regulation in the testis. A close comparison of previously published cytoplasmic effects on gene expression (Innocenti *et al.* 2011) and the results reported here suggest the two experiments yield qualitatively different results: our experiments affect the expression of more genes (compare Table 1 to Table S1); the magnitude of the gene expression effects we observe are larger (Table S2 and Figure S5); and the effects are more concentrated among testis-specific genes (Table S1 and Table S2; compare Figure S2 with Figure S6 and Figure S7). We therefore conclude that reversing sex chromosome parent-of-origin has gene expression effects above and beyond those that might be attributable solely to the cytoplasm.

### Opposing consequences of parent-of-origin effects in male and female progeny

The genes that display sensitivity to sex chromosome parent-of-origin inheritance in both sexes (Figure 4) suggest a shared regulatory effect in males and females. This effect cannot be attributable to cytoplasmic factors, because both female genotypes inherited the *Autw132* cytoplasm. Additionally, this effect is unlikely to be associated with imprinting of the X chromosome, because both female genotypes inherited both a paternal and a maternal X chromosome. These 41 genes therefore represent the most compelling evidence for Y-linked imprinting effects on global gene expression in our data. The small number of these genes, despite the extensive replication of the microarray experiments, is consistent with previous results that suggest imprinting is rare in adult female *Drosophila melanogaster* (Coolon *et al.* 2012; Wittkopp *et al.* 2006). The negative correlation between the gene expression effects of Y chromosome imprinting in males and females is reminiscent of the male-specific lethality associated with mutations in HP1 (Liu *et al.* 2005) and indicates that heterochromatic regulation of these genes leads to opposing expression changes (upregulation *vs.* downregulation) between the sexes. Additional experiments are required to ascertain whether such sex-specific heterochromatic effects are involved in the resolution of evolutionary conflicts over optimal expression between the sexes (Arnqvist and Rowe 2005; Stewart *et al.* 2010; Svensson *et al.* 2009).
